# Reduced amygdala habituation to anticipated social rejection in youth with major depressive disorder

**DOI:** 10.1016/j.jad.2026.121380

**Published:** 2026-02-16

**Authors:** M. Morningstar, M.N.K. Gravelle, D.P. Dickstein, J.S. Silk, R.E. Dahl, E.E. Nelson, D. Yee, L.R. Stroud

**Affiliations:** a Department of Psychology, Queen’s University, Canada; b Centre for Neuroscience Studies, Queen’s University, Canada; c Division of Child and Adolescent Psychiatry, McLean Hospital, USA; d Department of Psychology, University of Pittsburgh, USA; e Institute of Human Development, University of California Berkeley, USA; f Center for Biobehavioral Health, Nationwide Children’s Hospital, USA; g Department of Cognitive and Psychological Sciences, Brown University, USA; h Carney Institute for Brain Science, Brown University, USA; i Department of Psychiatry and Human Behavior, Warren Alpert Medical School of Brown University, USA; j Center for Behavioral and Preventive Medicine, The Miriam Hospital, USA

**Keywords:** Social learning, Depression, Adolescent, Peer evaluation, Neural response

## Abstract

In addition to its risks for morbidity and mortality, adolescent-onset depression is considered a chronic threat to youth’s socioemotional development due to its potential disruption of social learning processes. Prior work has determined that youth with depression, or at-risk of developing depression due to familial history, show atypical neural responses to social feedback by peers. To better understand whether adolescents at varying levels of risk for depression show differential neural indices of social *learning*, the current study interrogated trial-by-trial patterns of youth’s neural habituation to repeated social feedback. Seventy-six 10-to 17-year-old girls (22 diagnosed with major depressive disorder [MDD], 30 at familial risk but without MDD, 24 without MDD or familial risk) completed a chatroom task in which they repeatedly anticipated being accepted or rejected by “mean” (likely to reject) and “nice” (likely to accept) virtual peer confederates. Beta-series analyses revealed that the bilateral amygdala showed evidence of habituation to repeated social threat by the “mean” peer across groups. However, girls with MDD showed attenuated habituation in the right amygdala when anticipating feedback from the “mean” peers. Amygdala habituation was present (though attenuated) when anticipating social feedback from the “nice” peers, but did not vary by depression group. Findings identify a failure to habituate to anticipated social threat in MDD, suggesting altered social learning at the neural level. Attending to the dynamics of changing neural responses to anticipated social feedback may provide deeper insights into alterations in social and affective learning processes relevant to developing depression in adolescence.

The incidence of depression increases dramatically in adolescence (Brody and Hughes, 2025; Merikangas et al., 2010; Weinberger et al., 2018), particularly in girls (e.g., Avenevoli et al., 2015). Adolescent depression is considered more consequential than adult-onset depression (Kaufman et al., 2001) because of its negative effects on social functioning during a formative period of socio-emotional development. This highlights the importance of understanding developmental and learning processes relating to vulnerability to depressive symptoms during the teenage years. Theoretical frameworks for understanding adolescent depression have suggested that neurobiological sensitivity to socio-emotional information is a risk factor for the development of depressive symptoms (e.g., Forbes and Dahl, 2005; Forbes et al., 2021); one way this may confer risk is by interfering with social learning that occurs through interactions with peers (Kujawa and Burkhouse, 2017). Previous research has reported that depression (and familial risk for depression) is indeed associated with atypical neurobiological response to social evaluative feedback in youth (e.g., rejection or acceptance from peers; Freeman et al., 2022; Silk et al., 2014; Stroud et al., 2023; Stroud et al., 2017). However, less is known about how depression may interfere with neural indices of *social learning* (e.g., habituation of neural response to [social evaluative] threat; Yin et al., 2018). To better understand how social learning processes may be disrupted by depression, the current study leverages data from a chatroom task—during which youth receive repeated cues of acceptance and rejection from fictional virtual peers—to examine how neural response to repeated social feedback varies across time in adolescent girls with major depressive disorder (MDD), relative to girls without MDD who were at high or low depression risk based on familial history.

## 1.1. Neural response to social evaluative information in depression

Compared to typically developing adolescents, youth with depression and at-risk of developing depression due to familial histories show aberrant neural responses to social and emotional information (see reviews by Forbes et al., 2021; Kujawa and Burkhouse, 2017). In particular, the amygdala has been identified as a central node of socioemotional processing that is disrupted in depression. For instance, adults and youth at-risk of/experiencing depression show increased amygdala activation to negative emotional stimuli (e.g., Abler et al., 2007; Drevets, 2003; Hamilton et al., 2012; Monk et al., 2008; Peluso et al., 2009; Yang et al., 2010). Such variation in neural function in social-affective systems combined with experiences of social threat may be an important pathway to the emergence of depression in adolescence (Forbes et al., 2021). Indeed, differential neural processing of social feedback in particular has been linked to vulnerability for depression (Guyer, 2020; Kujawa and Burkhouse, 2017; Platt et al., 2013; Shields et al., 2024; Silk et al., 2023; Slavich et al., 2010).

The amygdala may be particularly relevant to social learning. This region has been conceptualized as a key node in networks involved in salience processing, as the amygdala is consistently found to be sensitive to salient information in socioemotional environments (see review by Adolphs, 2010) and to influence activation in regions related to detecting salience and prioritizing relevant information (e.g., insula; Craig, 2009; Ince et al., 2023). Importantly, several studies have found evidence of amygdala habituation following repeated presentations of emotional stimuli (e.g., emotional faces or negative images; Breiter et al., 1996; Denny et al., 2014; Fischer et al., 2003; Plichta et al., 2014; Wright et al., 2001)—potentially reflecting learned adaptation to stable patterns (Herry et al., 2007; Zald, 2003). Amygdala habituation to repeated emotional stimuli has been found to be attenuated in youth with internalizing disorders (van den Bulk et al., 2016) or individuals at risk for depression (Berhe et al., 2022), suggesting altered learning at the neurobiological level. Because adolescence is understood as a sensitive period for social learning (Blakemore and Mills, 2014; Dahl et al., 2018)—during which the amygdala is hypothesized to reorganize the attribution of salience to peer cues (Scherf et al., 2013)—disruptions of learning processes or an altered ability to adapt to stable patterns during this developmental stage may establish long-term patterns of amygdala hyperresponsivity (which may result in ongoing vulnerability to depression). However, while prior research has focused on risks associated with diminished amygdala habituation to repeated negative emotional stimuli (outside of social evaluative contexts), little attention has been given to how the amygdala habituates to repeated *social* threat in peer interactions—a situation that is commonly encountered during adolescence. Examining the temporal course of amygdala response to anticipated social threat may provide insight into whether *social* learning processes are altered in adolescents with, or at-risk of, depression.

## 1.2. Current study’s goals and hypotheses

The current study examined whether the time course of amygdala response to anticipated social rejection—a potent threat in adolescence—varied as a function of MDD risk in pre-adolescent and adolescent girls. To do so, we leveraged data from a sample of youth who had completed the “Chatroom Interact” task (involving the receipt of both positive and negative social feedback in interactions with peeraged fictional confederates; Silk et al., 2014) while undergoing functional magnetic resonance imaging (fMRI). Building upon prior work with this sample reporting that youth with/at-risk for MDD showed altered responses to being accepted or rejected (Stroud et al., 2023), we here examined neural activation during the *anticipation* of rejection. The task offers an interesting opportunity to examine social learning, as one of the virtual fictional peers (hereafter, “confederate”) is programmed to reject the participant 67% of the time and the other is programmed to select them 67% of the time for a discussion about various topics. Whether a confederate is rejecting or accepting isn’t readily apparent at first, but rather is learned over trials as the simulated peers repeatedly reject/accept the participant. The current study thus leveraged this experimental design and beta-series analysis (Yin et al., 2018) to determine whether amygdala activation while anticipating a given confederate’s decision a) varied over trials, as social learning occurs, and b) varied with depression status across groups.

We expected that the amygdala would habituate to anticipating the rejecting confederate’s response over the trials of the task, as this virtual peer’s behaviour is putatively learned. In line with previous literature suggesting reduced habituation to negative stimuli in youth with internalizing disorders (van den Bulk et al., 2016) and those at risk for depression (Berhe et al., 2022), we hypothesized that youth with MDD vulnerability (with either current MDD or familial risk for MDD) would show less habituation of amygdala activation over trials, compared to youth at low risk for MDD.

To probe the specificity of our findings, we also examined the temporal dynamics of neural response in other regions of interest (ROIs) relevant to processing threat and reward, *and* demonstrating altered function in depression, but not canonically involved in habituation: the anterior insula (AI) and subgenual anterior cingulate cortex (sgACC; Forbes et al., 2021; Hamilton et al., 2012; Kerestes et al., 2014; Miller et al., 2015). Prior research has implicated these regions in response to social rejection in typically developing adolescents (e.g., Masten et al., 2009; Sebastian et al., 2011), with evidence for heightened AI and sgACC response to rejecting feedback from peers in teens with depression (Davey et al., 2011; Jankowski et al., 2018; Silk et al., 2014). Moreover, response to social evaluative feedback in the sgACC predicts depressive symptoms prospectively (e.g., Masten et al., 2011; Silk et al., 2023), suggesting this region’s sensitivity to social cues is functionally meaningful for understanding *risk* for depression. The inclusion of these comparator ROIs was intended to determine the specificity of any findings pertaining to the amygdala; although other regions and networks may also be implicated in social information processing and depressive symptomatology, we strove for parsimony in our selection of appropriate comparison ROIs. Lastly, because the amygdala has also been implicated in processing reward salience (review in Pryce, 2018), we replicated our analysis in trials with the accepting confederate, to determine whether any differential patterns of habituation were specific to the risk of rejection or to *any* social evaluative component.

## 2. Method

### 2.1. Participants

Participants were recruited for the Child and Adolescent Thoughts in Social Situations (CHATSS) study from the local community (via flyers and digital posters), schools, and physician/mental health offices in the northeast United States. Seventy-six adolescents (all assigned female at birth), ages 10 to 17 (mean age = 13.41, *SD* = 2.35), were classified as part of a low-risk control group with no current/past personal or parental psychiatric disorder (*n* = 24), an at-risk group with a parental history of MDD (in one or both parents) but no current/past diagnosis of MDD themselves (*n* = 30), or a group with a current diagnosis of MDD (*n* = 22), based on semi-structured clinical research interviews (see Section 2.2.1). All procedures were approved by institutional ethics review boards. We obtained written parental consent and participant assent.

Based on self-report of racial identity, 55% of participants identified as (non-Hispanic) White, 20% as Latinx, 12% as Black, 1% as American Indian/Native Alaskan, 1% as Asian, and 11% as multiracial. Based on the Hollingshead four-factor index (Gottfried, 1985), 13.5% of the sample were of lower socioeconomic status (SES), 59.5% of middle SES, and 27% of high SES. Groups did not differ in race or SES (*p*s > .05) but differed in age (*p* = .014); age was thus entered as a control variable in analyses (see Section 2.4). A detailed description of sample characteristics can be found in Stroud et al. (2023).

### 2.2. Procedure

#### 2.2.1. Clinical interview

Screening and clinical assessments are described more fully in Stroud et al. (2023). No participant was diagnosed with a developmental condition (assessed during screening). Psychiatric history was obtained via clinical interviews. Youth participants completed the Kiddie Schedule for Affective Disorders and Schizophrenia in School-age Children Present and Lifetime version (K-SADS-PL; Kaufman et al., 1997). Primary caregivers completed the mood and anxiety modules of the Structured Clinical Interview for the DSM-IV (SCID; First, 2002); 43% of secondary caregivers also completed the SCID, with parental history for the remaining 57% provided by primary parent report. Reliability was high for both current and lifetime MDD (based on second coding by a licensed clinician; kappa = 1.0 for current MDD, 0.86 for youth lifetime MDD, 0.83 for parent lifetime MDD). No participant had a parental or personal history of psychosis or bipolar disorder. Three participants in the MDD group were on a stable dose of antidepressant medication; 1 was taking a stimulant (not taken on the day of the MRI scan). No other participants (in any group) were taking medication regularly.

Beyond MDD diagnoses, 10/22 participants in the MDD group met criteria for an additional current diagnosis, including generalized anxiety disorder (*n* = 8), social phobia (*n* = 1), obsessive compulsive disorder (*n* = 1), and attention-deficit/hyperactivity disorder (ADHD; *n* = 1). In addition, 4/30 participants in the at-risk group met criteria for non-MDD or mood-related current diagnoses: these included generalized anxiety disorder (*n* = 3), separation anxiety (*n* = 1), and ADHD (*n* = 1). None of the participants in the low-risk group met criteria for any past or current psychiatric diagnosis.

#### 2.2.2. fMRI Chatroom task

At the end of the interview session, all participants were asked to choose both 5 female and 5 male youth (from 30 profiles and photographs of similar aged peers from the National Institute of Mental Health’s Child Emotional Faces Picture Set; Egger et al., 2011) that they would be interested in chatting with remotely during the second study visit. They also provided a photograph and information for their own profile for the putative other youth to view. In the second visit (median 1.5 weeks later), participants completed the Chatroom Interact task (Silk et al., 2014; modified to be a rapid event-related design) while undergoing fMRI.

During each run of the Chatroom Interact task, all participants were “connected” to two other youth (two girls in the first run of the task, and two boys in the second). They were told that each person in the chatroom would take turns to select one person with whom to discuss 15 different topics (e.g., friends, books, music). For each topic, the selected person’s profile was highlighted (“selected”) and the other person’s profile was covered with an “X” (“rejected”). The participant selected their topic partners first. Following this, each confederate selected their topic partners (during which time participants were asked to identify via button press who had been selected, to maintain task engagement). In each run, one of the confederates rejected the participant 67% of the time (hereafter, “mean” confederate); the other confederate rejected the participant 33% of the time (hereafter, “nice” confederate). The outcome was randomized within confederate. Over the 60 trials of the task (2 runs × 2 confederates × 15 topics), participants were rejected in 30 trials (50%) and selected in 30 trials (50%). Each trial was separated into an anticipation period, in which the confederate is making their choice but the result has not been displayed yet (only pictures of the confederates are displayed), and the outcome period (i.e., “selected” or “rejected”). Each of these periods was 2500–6250 ms in duration (*M* duration = 4750 ms) to mimic a real Chatroom in which other participants’ responses would likely vary in duration. The current study focuses on the anticipation periods, during which activation may be expected to change across trials as participants learn who is most likely to reject them.

After the task was complete, participants rated their interest in the task and how they felt (excluded, included, happy, sad, nervous, angry) when being selected and rejected by the confederates, on 10-point Likert scales (from “Not at all” to “Extremely”; reported in Stroud et al., 2023). No participant reported suspicion about the study procedure or confederates. Participants were informed of the deception during debriefing.

### 2.3. Image acquisition and preprocessing

MRI data were acquired between July 2012 and September 2015 on a 3 Tesla Tim Trio scanner. A T1-weighted anatomical scan (MPRAGE) was first obtained with 1 mm isometric voxels, 160 sagittal slices, repetition time (TR) = 2250 ms, echo time (TE) = 2.98 ms, and field of view (FOV) = 256 × 256 mm. Echo planar imaging (EPI) scans were then obtained over two functional runs, with 3 mm isometric voxels, 40 slices, TR = 2500 ms, TE = 28 ms, and FOV = 192 × 192 mm.

EPI images were preprocessed and analyzed in AFNI (Cox, 1996; version 18.1.05), according to standardized pipelines. Images were aligned to the first volume, oriented to the AC/PC line, co-registered to the anatomical image, and warped non-linearly to the Talairach template. Images were spatially smoothed (Gaussian filter, FWHM 6 mm) and voxel-wise signal was scaled within-participant to a mean value of 100. Volumes with more than 10% of voxels tagged as signal outliers (over 200) or with movement greater than 1 mm from the next volume were censored at the subject-level. Nuisance regressors for motion (6 affine directions and their first-order derivatives) and scanner drift (within each run) were also included at the subject level.

To examine change in amygdala response over repeated rejections by a peer, we conducted beta-series analyses of trial-by-trial activation (Yin et al., 2018) during anticipation periods. Each anticipation period is treated as a regressor at the subject-level (modeled as 4750 ms in duration to ensure consistent amounts of data were contributing to estimates of BOLD response across trials of the task), yielding a beta value for each trial. Beta values were extracted from the left and right amygdala ROIs, as well as the comparator AI and sgACC ROIs. ROIs were derived from the Talairach-Tournoux atlas in AFNI (using the BA25 mask for the sgACC); masks were dilated by 3 voxels and eroded by 1 voxel before being applied (see Supplemental materials for a visualization of all ROIs).

Beta values from each ROI were then submitted to growth curve models (see Section 2.4). Trials for which more than 1 volume was censored for motion (1.16% of total trials) or flagged as signal outliers (0.15% of trials), and blocks in which more than 20% of volumes were censored for motion (12 blocks from 10 participants; 3.95% of trials), were removed from analyses (total of 5.26% of all trials). Growth curve models were fit to 4320 trial-level betas (2211 in “mean” trials, 2109 in “nice” trials) from 76 participants.

### 2.4. Statistical analysis

Growth curve models (Mirman, 2014) were fit to the amygdala’s betas across the 15 trials (per run) in which the “mean” confederate was the decision-maker, using *lmerTest* (Kuznetsova et al., 2017) in R (version 2024.04.2; PositTeam, 2024). The slope of amygdala activation over trials was modeled with second-order orthogonal polynomials (capturing linear and quadratic trends across trials) with a fixed effect of MDD status group (3 levels; low-risk controls as the reference group). Per-participant random intercepts and slopes (linear and quadratic) were included in the model to account for individual variability in neural response to the task. Mean-centered age (in years) was entered as a control variable, given differences in age across depression groups. The model was fit by maximum likelihood, using a *bobyqa* (Bound Optimization BY Quadratic Approximation) optimizer. *P* values for fixed effects were obtained using Satterthwaite’s approximation (using the *summary* function in R).

We then replicated the above analysis with the amygdala’s betas across the 15 trials (per run) in which the “nice” confederate was the decision-maker, to determine whether habituation patterns generalized outside of the context of repeated social threat. Lastly, to probe the specificity of these effects, the above models were also fit to betas in the comparator ROIs (i.e., AI, sgACC). We retained α = .05 for these additional analyses, as they were intended to test the specificity of the primary analysis (i.e., amygdala response to potential social threat). Analysis code is available on the Open Science Framework (OSF): https://osf.io/f2nx8/?view_only=fcf7b704e0454e1bb275a159e8b9fab3.

## 3. Results

**Fig. 1.**
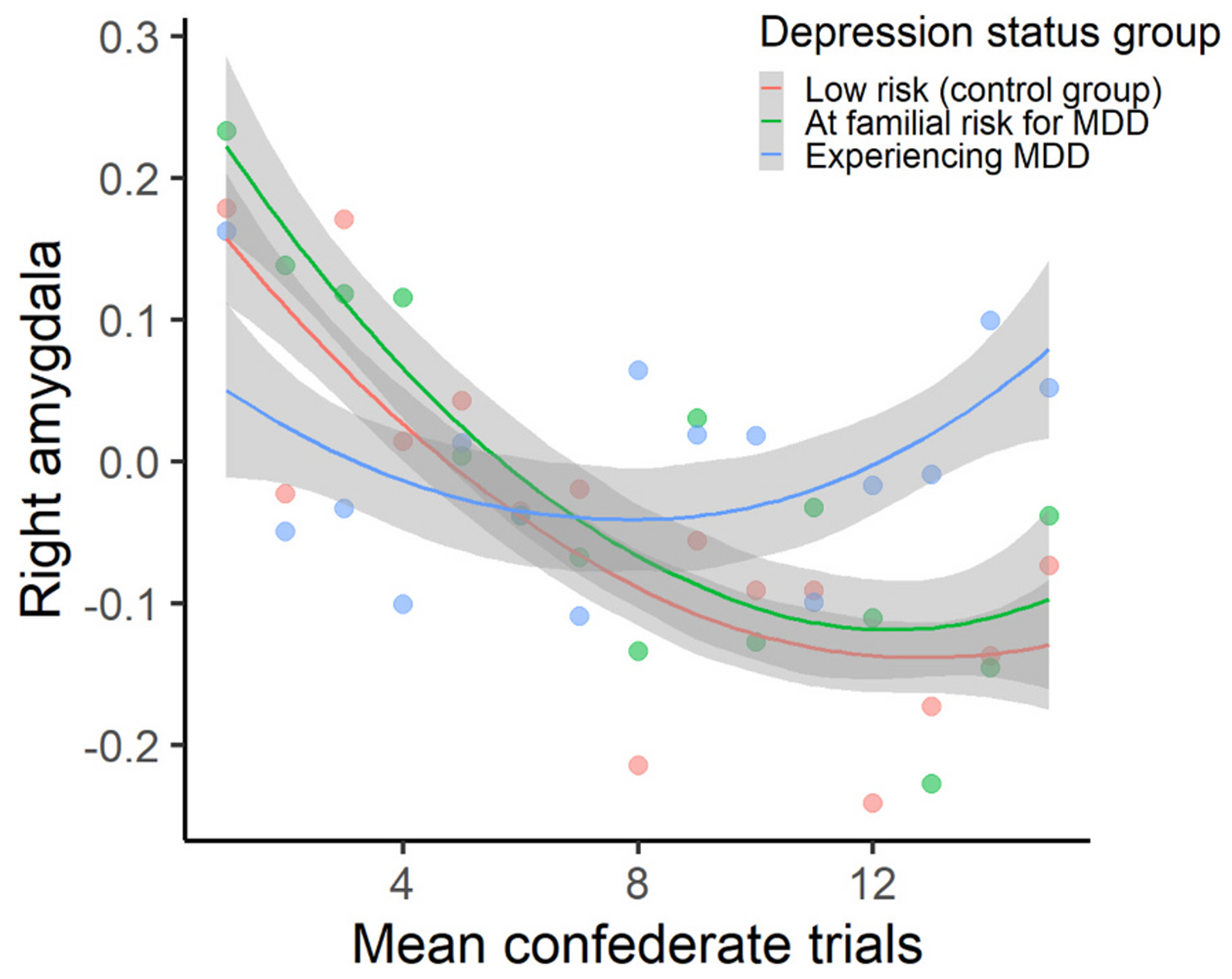
Amygdala habituation to anticipated rejection by depression status group. Note. Right amygdala activation across 15 trials of anticipating the “mean” (i.e., more likely to reject) confederate’s decision in the Chatroom task. Solid lines represent the trend line for amygdala activation across trials, by depression status group; dots represent the average amygdala response at each trial, for each group (averaged across participants in a given depression status group); shaded ribbon represents the standard error of the mean.

There were no overall differences between MDD status groups in the magnitude of amygdala activation in anticipation of “mean” confederates’ decisions (*p*s > .650). For both the left and right amygdala, activation decreased linearly across trials with “mean” confederates (left: *p* = .019, right: *p* = .008; Table 1). In the right amygdala, this slope varied by MDD status (*p* = .040): compared to low-risk controls,^1^ girls with MDD showed a flatter slope of amygdala activation across trials (Fig. 1), suggesting reduced habituation to anticipating “mean” confederates’ decisions over time. Control analyses with the “nice” confederate trials (Table 2) did not replicate this pattern, suggesting specificity to the social context of heightened rejection threat. Although the U-shaped quadratic pattern of amygdala activation across trials with “nice” confederates (left: *p* = .005, right: *p* = .042; see Figure in Supplemental materials) suggests attenuated habituation when rejections are less frequent (i.e., initial decrease in response but also reactivation in later trials), this pattern did not vary across MDD groups (*p*s *>* .133).

In the comparator ROIs (Supplemental Table 1), there was evidence of habituation in the left and right AI across “mean” confederate trials (linear negative slopes across trials; *p*s = .002), but not across “nice” confederate trials (*p*s > .305). At-risk girls showed heightened activation in the left sgACC when anticipating the “mean” confederates’ decisions compared to the low-risk controls (*p* = .015), but not the “nice” confederates’ decisions. The slope of activation in these ROIs did not vary by MDD status group (*p*s > .081).

## 4. Discussion

Using beta-series analysis (e.g., Yin et al., 2018), we examined the temporal dynamics of neural activation during the anticipation of peer feedback across trials with rejecting/“mean” confederates (i.e., in a high rejection context), in the amygdala and in comparator regions of interest associated with processing threat and reward/salience (anterior insula and subgenual anterior cingulate cortex; Forbes et al., 2021). Girls with MDD—but importantly, not those at familial risk for MDD—showed temporally stable activation of the right amygdala when anticipating social rejection. This failure to habituate may index altered social learning in youth with MDD. Given findings that peer rejection is associated with depressive symptoms in youth (e.g., Hawker and Boulton, 2000; Platt et al., 2013; Zwierzynska et al., 2013)—and that neural response to social feedback mediates that association (Pagliaccio et al., 2023)—our findings highlight the importance of investigating atypical dynamics of neural response to social threat as a mechanism through which peer experiences may shape neurobiological sensitivities to predict depression in adolescents.

### 4.1. MDD was associated with attenuated amygdala habituation to potential social threat

Adolescent girls who were *not* experiencing MDD—regardless of familial history of MDD—showed amygdala habituation in anticipation of repeated potential social threat. As the rejecting/“mean” peers’ likelihood to reject became more apparent over the trials of the task, both groups showed linear decreases in bilateral amygdala activation. A similar pattern was also noted in the bilateral insula, consistent with prior findings of insula habituation to emotional faces (Feinstein et al., 2002; Plichta et al., 2014) and its involvement in social learning (Olsson et al., 2020). However, girls with MDD showed temporally stable activation of the right amygdala when anticipating rejecting peers’ evaluative feedback (but no habituation differences observed in either the AI or sgACC). This pattern may be interpreted as a specific failure of the amygdala to habituate to the potential threat these peers posed over time.

These findings are consistent with prior work showing that amygdala habituation to repeated emotional faces in typically developing samples (Breiter et al., 1996; Fischer et al., 2003; Wright et al., 2001) is attenuated in depression (van den Bulk et al., 2016). Importantly, our findings demonstrate that habituation principles are also at play in contexts of repeated social threat. Strikingly, this pattern was present when *anticipating* the rejecting peers’ feedback, and not to actual threat/ negative emotional faces (as both the “nice” and “mean” conditions featured the smiling faces of the putative peers). Moreover, findings of altered neural processing in depression were not noted in anticipating social acceptance from the accepting/“nice” confederates (similar to Silk et al., 2014). Habituation is less robust in response to positive stimuli (e. g., Breiter et al., 1996; Wright et al., 2001) and was attenuated when anticipating feedback from the accepting peer across all groups (i.e., quadratic change in amygdala activation over trials rather than linear decrease). Thus, while habituation to anticipated social feedback can occur in both accepting and rejecting peer contexts, only response to potential threat was potentiated by MDD.

This pattern was not noted in girls at-risk for MDD, suggesting that invariable amygdala sensitivity to potential social threat may be associated with the experience of/current MDD, but not necessarily familial risk for MDD alone. Instead, at-risk girls showed greater activation in the left sgACC than low-risk girls when anticipating the rejecting peers’ decisions; our prior work showed that the same group of youth showed heightened response in this region to the *receipt* of social feedback as well (Stroud et al., 2023), denoting sgACC activation as a potential marker of enhanced engagement with social evaluative information overall in at-risk youth.

### 4.2. Social learning and salience processing in adolescent MDD

Altered neural processing of interpersonal interactions may represent a form of neurobiological susceptibility to social rejection that could compound difficulties in social functioning in youth with MDD (see reviews by Forbes et al., 2021; Guyer, 2020; Slavich et al., 2010). For instance, a failure to habituate may represent a failure to regulate or update response adequately to new information about others. The basolateral amygdala in humans is thought to be involved in “social experiential learning” (as lesions to this area impair the ability to adapt behaviour in response to others’ choices in cooperative games; Rosenberger et al., 2019). Amygdala response is typically relevant to learning new patterns (e.g., object-emotion associations; Hooker et al., 2006): activation is highest when forming new associations or in the context of unpredictable stimuli (Herry et al., 2007), but decreases once contingencies are learned/information is predictable (Büchel et al., 1998; Herry et al., 2007). As new information about the rejecting peers is acquired over trials, the learned probability of being rejected may yield amygdala habituation when anticipating these peers’ decisions in girls without MDD. A new trial does not necessarily represent new information about these peers, as their past behaviour is sufficient to determine the likelihood of being rejected. In contrast, girls with MDD may have failed to learn the association between the “mean” peers and the likelihood of social rejection, or retained an unchanging expectation of being rejected—yielding invariable amygdala activation in anticipation of their decisions well into the task.

Importantly, the amygdala does not act alone: it is a central node in salience-related networks (Redcay and Warnell, 2018) that modulates activation in functionally connected regions involved in attending to, remembering, and interpreting social information (e.g., anterior insula, dorsal anterior cingulate [Ince et al., 2023], hippocampus [Zheng et al., 2017]). Our findings of depression-related differences in habituation in the amygdala *but not* the anterior insula highlight a potential disconnect between nodes of the salience network in MDD. Indeed, adolescentonset MDD has been associated with a decoupling of the amygdala from the salience network (Jacobs et al., 2016) and an expansion of the cortical size of the salience network (Lynch et al., 2024), suggesting that deviations in social learning (e.g., invariable amygdala activation to potential threat) may impact *salience processing* more broadly over time. The iterative sculpting of neural response to prioritize salient threats of rejection may then influence social behaviours (e.g., heightened vigilance and emotional response to cues of rejection, withdrawal from social situations) that could contribute to the symptomatology of MDD (e.g., Katz et al., 2011)—although bidirectional influences are possible (i.e., the experience of MDD itself could lead to altered habituation processes at the neural level). Altogether, these findings point to potential mechanisms through which salience processing can shape, and be shaped by, social learning—in ways that can impact teenagers’ psychosocial adjustment.

Attending to the *neural dynamics* of youth’s response to social evaluative feedback in brain regions associated with processing salience and affective cues can provide important insights into how depression may alter social learning processes in youth. Depression risk has been conceptualized as a dynamic process whereby socio-affective neural systems interface with social cues in dynamic contexts (Forbes et al., 2021). Thus, leveraging tools to examine differences in the time course of physiological, neural, and behavioural responses to social evaluative feedback across risk groups will allow us to interrogate how social experience with peers interacts with neurobiological sensitivities, at varying timescales (e.g., short-term learning within a task, or long-term learning in longitudinal designs). Recent work utilized trial-by-trial data in a Chatroom paradigm to estimate youth’s fluctuating neural coding of peers in a social evaluation context (Kumar et al., 2019); integrating this kind of method, and capitalizing on known neural phenomena associated with learning (e.g., habituation), holds promise for further understanding of how differential sensitivity to peer feedback may result in vulnerability for depression.

### 4.3. Strengths and limitations

The current study leveraged trial-by-trial data to determine that neural habituation to repeated stimuli—typically investigated in conditioning or face emotion matching paradigms (e.g., Plichta et al., 2014; Yin et al., 2018)—was also noted in anticipation of repeated social threat, and distinguished youth with and without MDD. By including non-depressed youth at familial risk for MDD, we expanded our understanding of vulnerability to MDD in youth—an area of research that was noted to be sparse in a recent review (Kujawa and Burkhouse, 2017). Moreover, our findings highlight the promise of investigating the time course of neural response as an index of social learning, to better understand the mechanisms through which differential neural sensitivity to social evaluative feedback may increase risk for MDD in youth.

Limitations must be noted. First, our modest sample size—while similar to that of comparable fMRI studies of adolescent depression—precluded well-powered investigations of additional interindividual differences that may have impacted the slope of habituation [e.g., rejection distress (Masten et al., 2009), social fearfulness (Avery and Blackford, 2016), anxiety (Hare et al., 2008)]. Probing predictors of habituation dynamics may help identify youth most at risk of altered social learning. Future directions would also benefit from a neural network-oriented approach, considering the dynamics of activation in a broader set of interconnected regions involved in processing negative social feedback (e.g., dorsal anterior cingulate cortex; Dedovic et al., 2016) and how they may be altered in groups at varying levels of risk for depression.

Second, the current study focused on adolescent girls, who are at heightened risk for depression at this age (e.g., Avenevoli et al., 2015) and show heightened neural sensitivity to anticipated peer evaluation (e.g., Guyer et al., 2009); however, as a result, we are unable to determine whether similar social learning processes are at play in youth with other gender identities at this age or for any gender in adulthood. Lastly, we employed common conceptualizations of habituation to make inferences about learning (Adolphs, 2010; Gallagher and Holland, 1994; Olsson et al., 2020), but did not measure explicit learning in the context of this task. Verifying whether habituation at the neural level influences trial-by-trial behavioural ratings of peers, narrative impressions of social risk (e.g., whether the “mean” peer was identified as such by participants), or real-world social behaviours, is an important future direction to validate neural habituation as an index of social learning.

## 5. Conclusion

We report evidence for attenuated amygdala habituation to repeated anticipated social threat in girls experiencing MDD, but not girls without MDD (with or without familial risk). Amygdala habituation was present (but attenuated) when anticipating social acceptance, and did not vary by MDD status. Extending previous findings of altered neural response to peer feedback in youth with/at risk for MDD, we contend that attending to the *dynamics of neural response to social evaluation* is a promising avenue for future research on social learning processes in adolescent depression.

## Supplementary Material

Supplemental Material

## Figures and Tables

**Table 1:** Parameter estimates for amygdala response across “mean” confederate trials.

	Left amygdala			Right amygdala		
Effect	*b* (SE)	95% CI	*p*	*b* (SE)	95% CI	*p*
ot1	−0.295 (0.124)	[−0.537, −0.053]	.019*	−0.348 (0.127)	[−0.596, −0.099]	.008**
ot2	0.096 (0.118)	[−0.136, 0.328]	.418	0.135 (0.105)	[−0.071, 0.342]	.203
Depression (at-risk)	−0.024 (0.080)	[−0.180, 0.133]	.768	0.035 (0.076)	[−0.115, 0.185]	.650
Depression (MDD)	−0.026 (0.087)	[−0.196, 0.144]	.763	0.028 (0.083)	[−0.135, 0.191]	.737
Age	−0.003 (0.013)	[−0.030, 0.023]	.809	−0.001 (0.013)	[−0.027, 0.025]	.940
ot1 × Depression (at-risk)	−0.051 (0.165)	[−0.375, 0.273]	.757	−0.034 (0.170)	[−0.366, 0.298]	.842
ot1 × Depression (MDD)	0.209 (0.179)	[−0.141, 0.560]	.246	0.384 (0.184)	[0.024, 0.745]	.040*
ot2 × Depression (at-risk)	0.212 (0.158)	[−0.098, 0.522]	.185	0.031 (0.141)	[−0.245, 0.306]	.828
ot2 × Depression (MDD)	0.155 (0.172)	[−0.181, 0.491]	.369	0.035 (0.153)	[−0.265, 0.334]	.822

Note. ot1 = term for linear change over trials of a given confederate; ot2 = term for quadratic change over trials. Depression represents the ‘depression status’ predictor (MDD = major depressive disorder); the reference level for this predictor is the low-risk (healthy control) group. Age (in years) was mean-centered. *p* values are derived from Satterthwaite’s approximation. *b* = parameter estimate; SE = standard error; 95% confidence interval (CI). Bolded values are significant at α = .05.

* *p* < .05,

** p < .01.

**Table 2:** Parameter estimates for amygdala response across “nice” confederate trials.

	Left amygdala			Right amygdala		
Effect	*b* (SE)	95% CI	*p*	*b* (SE)	95% CI	*p*
ot1	0.157 (0.126)	[−0.089, 0.404]	.215	0.025 (0.142)	[−0.253, 0.303]	.861
ot2	0.300 (0.102)	[0.100, 0.500]	.005**	0.186 (0.089)	[0.011, 0.361]	.042
Depression (at-risk)	0.043 (0.066)	[−0.087, 0.173]	.515	0.059 (0.063)	[−0.064, 0.181]	.352
Depression (MDD)	0.082 (0.072)	[−0.059, 0.222]	.258	0.110 (0.068)	[−0.023, 0.243]	.110
Age	0.003 (0.010)	[−0.017, 0.022]	.798	−0.018 (0.011)	[−0.040, 0.004]	.120
ot1 × Depression (at-risk)	−0.081 (0.168)	[−0.410, 0.249]	.633	−0.040 (0.190)	[−0.412, 0.333]	.836
ot1 × Depression (MDD)	−0.026 (0.181)	[−0.381, 0.328]	.884	0.085 (0.205)	[−0.316, 0.486]	.680
ot2 × Depression (at-risk)	−0.176 (0.136)	[−0.443, 0.092]	.202	−0.067 (0.119)	[−0.300, 0.167]	.578
ot2 × Depression (MDD)	−0.224 (0.147)	[−0.512, 0.064]	.133	−0.138 (0.128)	[−0.390, 0.114]	.286

Note. ot1 = term for linear change over trials of a given confederate; ot2 = term for quadratic change over trials. Depression represents the ‘depression status’ predictor (MDD = major depressive disorder); the reference level for this predictor is the low-risk (healthy control) group. Age (in years) was mean-centered. *p* values are derived from Satterthwaite’s approximation. *b* = parameter estimate; SE = standard error; 95% confidence interval (CI). Bolded values are significant at α = .05.

* *p* < .05,

** *p* < .01.

## Data Availability

De-identified data are available from the first author upon reasonable request. Analysis code is available on OSF: https://osf.io/f2nx8/?view_only=fcf7b704e0454e1bb275a159e8b9fab3.

## Footnotes

1 An exploratory analysis revealed that relevelling the MDD status group to set the at-risk group as the reference category did not change results: the slope of right amygdala activation across trials also differed between the at-risk and MDD groups (*p* =.019).

## Appendix A. Supplementary data

Supplementary data to this article can be found online at https://doi.org/10.1016/j.jad.2026.121380.
