# Supplementary material for: Reduced amygdala habituation to anticipated social rejection in youth with major depressive disorder
Source: J Affect Disord. Author manuscript; Available in PMC 2026 Jun 13. (PMC13264236; doi:10.1016/j.jad.2026.121380)
Supplement: Supplemental Material [file NIHMS2166481-supplement-Supplemental_Material.docx]

Table S1. Parameter estimates for control ROIs across “mean” and “nice” confederate trials

|  | Left AI | | | Right AI | | | Left sgACC | | | Right sgACC | | |
| --- | --- | --- | --- | --- | --- | --- | --- | --- | --- | --- | --- | --- |
| Effect | *b* (SE) | 95% CI | *p* | *b* (SE) | 95% CI | *p* | *b* (SE) | 95% CI | *p* | *b* (SE) | 95% CI | *p* |
| **“Mean” confederate trials** | | | | | | | | | | | | |
| ot1 | -0.209 (0.066) | [-0.338, -0.079] | **.002 **** | -0.249 (0.076) | [-0.397, -0.100] | **.002 **** | -0.298 (0.203) | [-0.697, 0.101] | .147 | -0.058 (0.224) | [-0.498, 0.381] | .796 |
| ot2 | -0.013 (0.061) | [-0.133, 0.107] | .837 | -0.029 (0.066) | [-0.159, 0.101] | .659 | 0.021 (0.150) | [-0.272, 0.314] | .890 | 0.096 (0.154) | [-0.206, 0.399] | .533 |
| Depression (at-risk) | -0.012 (0.041) | [-0.093, 0.069] | .770 | 0.003 (0.044) | [-0.083, 0.089] | .948 | 0.255 (0.102) | [0.055, 0.455] | **.015  *** | 0.139 (0.092) | [-0.041, 0.320] | .135 |
| Depression (MDD) | -0.078 (0.045) | [-0.166, 0.010] | .088 | -0.048 (0.048) | [-0.142, 0.045] | .312 | -0.020 (0.111) | [-0.238, 0.198] | .860 | -0.079 (0.100) | [-0.275, 0.118] | .436 |
| Age | 0.001 (0.007) | [-0.014, 0.015] | .935 | 0.002 (0.008) | [-0.013, 0.018] | .777 | 0.032 (0.018) | [-0.004, 0.068] | .087 | 0.007 (0.016) | [-0.025, 0.038] | .680 |
| ot1 x Depression (at-risk) | -0.015 (0.088) | [-0.188, 0.158] | .869 | -0.089 (0.101) | [-0.287, 0.109] | .381 | <0.001 (.272) | [-0.533, 0.534] | .999 | -0.058 (0.300) | [-0.647, 0.530] | .846 |
| ot1 x Depression (MDD) | 0.120 (0.096) | [-0.068, 0.307] | .216 | 0.144 (0.110) | [-0.071, 0.359] | .193 | 0.400 (0.295) | [-0.178, 0.978] | .179 | 0.074 (0.325) | [-0.563, 0.711] | .821 |
| ot2 x Depression (at-risk) | 0.097 (0.082) | [-0.064, 0.257] | .241 | 0.121 (0.089) | [-0.053, 0.295] | .176 | 0.053 (0.200) | [-0.339, 0.445] | .793 | 0.170 (0.206) | [-0.234, 0.574] | .412 |
| ot2 x Depression (MDD) | 0.106 (0.089) | [-0.068, 0.281] | .236 | 0.151 (0.096) | [-0.038, 0.340] | .123 | 0.226 (0.217) | [-0.200, 0.652] | .303 | 0.396 (0.224) | [-0.043, 0.835] | .081 |
| **“Nice” confederate trials** | | | | | | | | | | | | |
| ot1 | -0.078 (0.076) | [-0.226, 0.070] | .305 | -0.060 (0.085) | [-0.228, 0.107] | .484 | 0.207 (0.166) | [-0.119, 0.532] | .217 | 0.144 (0.195) | [-0.238, 0.526] | .461 |
| ot2 | -0.012 (0.061) | [-0.133, 0.108] | .843 | 0.039 (0.068) | [-0.093, 0.172] | .565 | 0.081 (0.177) | [-0.265, 0.427] | .649 | 0.205 (0.189) | [-0.165, 0.575] | .282 |
| Depression (at-risk) | -0.009 (0.038) | [-0.083, 0.065] | .810 | 0.002 (0.044) | [-0.084, 0.089] | .956 | 0.051 (0.108) | [-0.161, 0.262] | .639 | -0.020 (0.121) | [-0.256, 0.217] | .872 |
| Depression (MDD) | -0.049 (0.041) | [-0.129, 0.030] | .229 | 0.001 (0.048) | [-0.093, 0.095] | .983 | 0.141 (0.117) | [-0.088, 0.370] | .233 | 0.037 (0.131) | [-0.219, 0.293] | .779 |
| Age | 0.005 (0.007) | [-0.008, 0.018] | .488 | 0.003 (0.008) | [-0.011, 0.018] | .664 | -0.011 (0.016) | [-0.043, 0.022] | .520 | 0.007 (0.018) | [-0.027, 0.042] | .685 |
| ot1 x Depression (at-risk) | 0.134 (0.101) | [-0.064, 0.332] | .188 | 0.068 (0.114) | [-0.156, 0.292] | .556 | -0.183 (0.222) | [-0.619, 0.252] | .412 | -0.135 (0.261) | [-0.646, 0.376] | .606 |
| ot1 x Depression (MDD) | 0.122 (0.109) | [-0.091, 0.336] | .264 | 0.099 (0.123) | [-0.142, 0.340] | .424 | -0.357 (0.239) | [-0.826, 0.111] | .139 | -0.054 (0.281) | [-0.604, 0.496] | .848 |
| ot2 x Depression (at-risk) | -0.056 (0.082) | [-0.217, 0.104] | .494 | -0.063 (0.090) | [-0.240, 0.115] | .491 | -0.077 (0.236) | [-0.539, 0.386] | .746 | -0.022 (0.252) | [-0.516, 0.473] | .932 |
| ot2 x Depression (MDD) | 0.038 (0.088) | [-0.135, 0.211] | .667 | -0.025 (0.097) | [-0.215, 0.166] | .800 | -0.236 (0.254) | [-0.734, 0.262] | .357 | -0.231 (0.272) | [-0.764, 0.302] | .398 |

*Note*. ot1 = term for linear change over trials of a given confederate; ot2 = term for quadratic change over trials. Depression represents the ‘depression status’ predictor (MDD = major depressive disorder); the reference level for this predictor is the low-risk (healthy control) group. Age (in years) was mean-centered. *p* values are derived from Satterthwaite’s approximation. *b* = parameter estimate; SE = standard error; 95% confidence interval (CI). Bolded values are significant at α = .05. * *p* < .05, ** p < .01.

**Figure S1.** Amygdala response to anticipated decisions by “nice” confederates


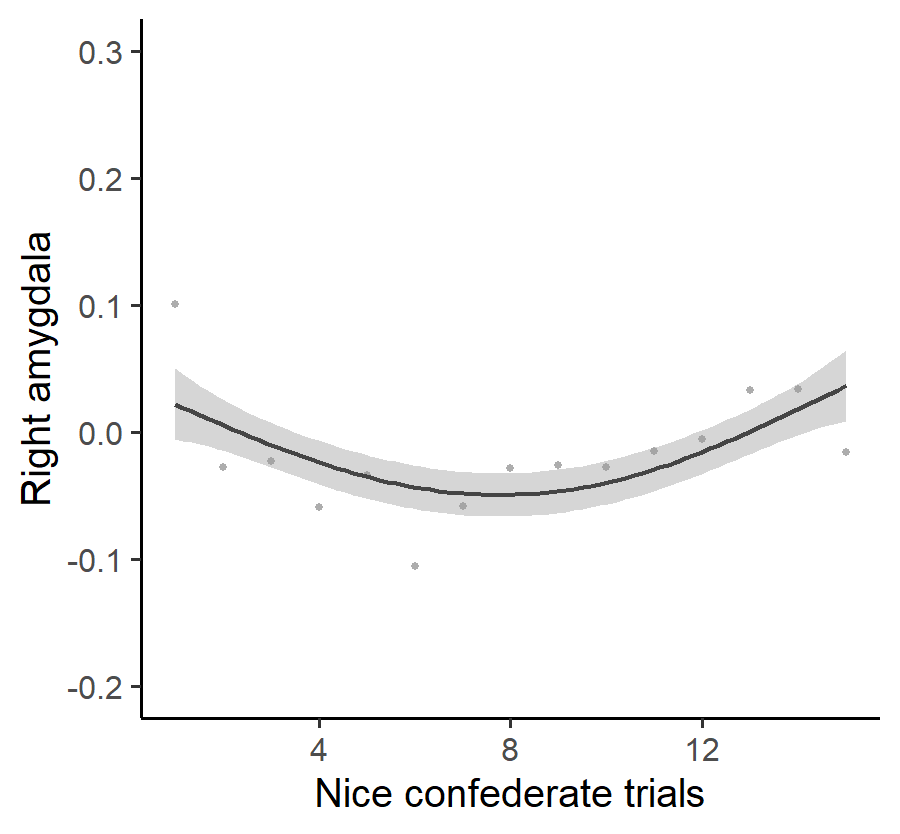

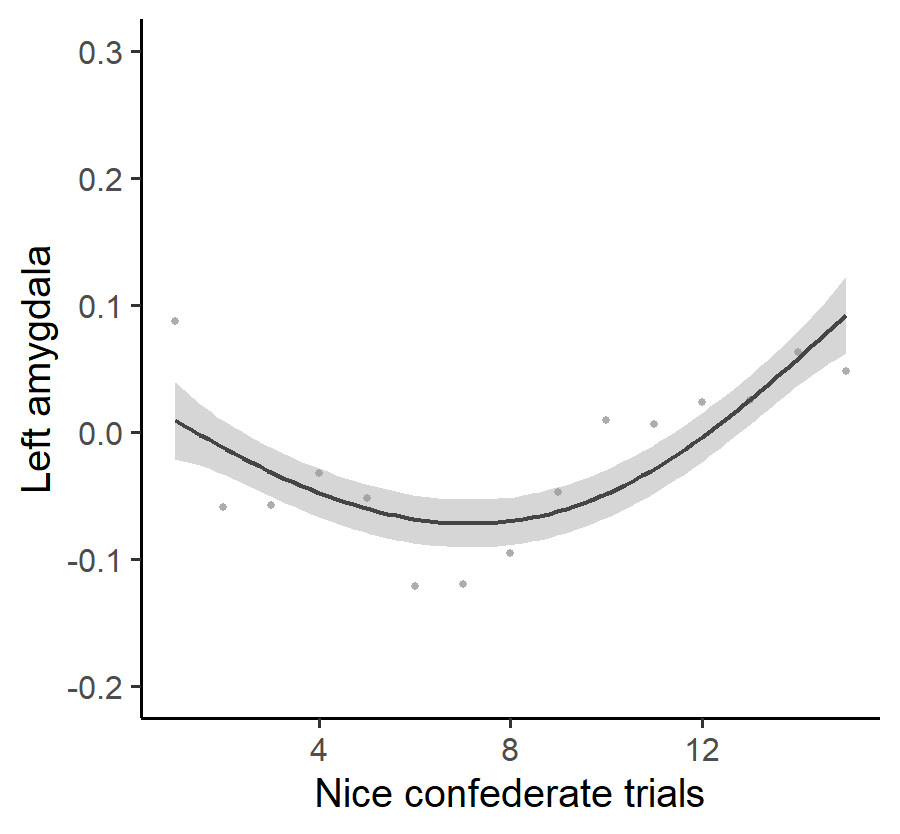


Note. Right and left amygdala activation across 15 trials of anticipating the “nice” (i.e., more likely to select) confederate’s decision in the Chatroom task. Solid lines represent the trend line for amygdala activation across trials; dots represent the average amygdala response at each trial (averaged across all participants); shared ribbon represents the standard error of the mean.

**Figure S2.** Amygdala and comparator regions of interest

**Comparator ROIs**

**Amygdala**


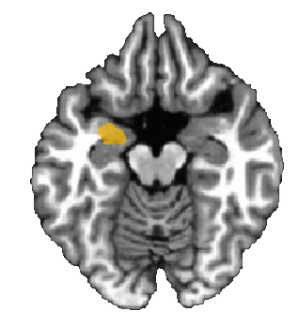

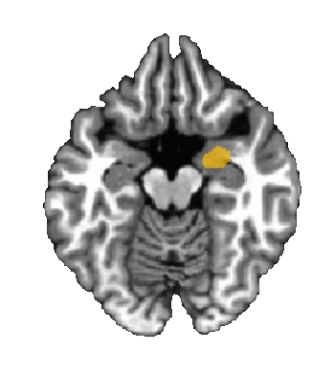


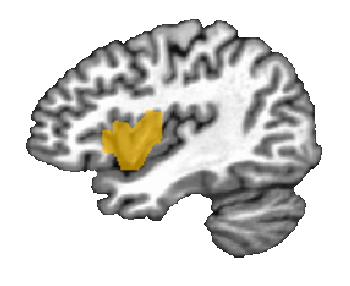

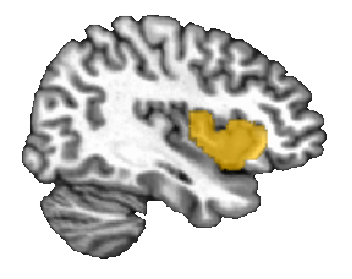


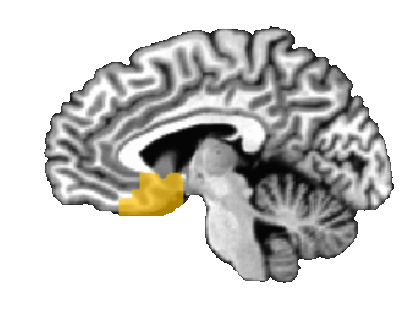

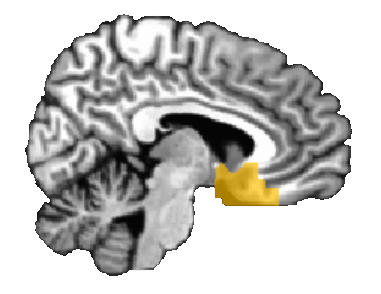


Note. Masks for left and right amygdala (left panel) and comparator ROIs (regions of interest; right panel). Comparator ROIs included the left and right anterior insula (AI; top row) and subgenual anterior cingulate cortex (sgACC; bottom row).
